# Laparoscopic Fenestration for Abscess Following SpaceOAR Placement—Case Report

**DOI:** 10.1002/iju5.70109

**Published:** 2025-11-11

**Authors:** Yuki Matsuo, Kimihiro Shimatani, Toeki Yanagi, Yusuke Yamada, Xiu‐Xian Wu, Akihiro Kanematsu, Masayuki Fujiwara, Hitomi Suzuki, Koichiro Yamakado, Shingo Yamamoto

**Affiliations:** ^1^ Department of Urology Hyogo Medical University Nishinomiya Japan; ^2^ Department of Urology Meiwa Hospital Nishinomiya Japan; ^3^ Department of Radiology Hyogo Medical University Nishinomiya Japan

**Keywords:** abscess, prostate cancer, SpaceOAR

## Abstract

**Introduction:**

SpaceOAR is a hydrogel spacer widely used to protect the rectum from radiation toxicity during prostate cancer treatment. Reported here is a case in which an abscess that developed following SpaceOAR placement was successfully treated with laparoscopic fenestration.

**Case Presentation:**

A 70‐year‐old man with localized prostate cancer undergoing a radiation therapy course developed an abscess at the SpaceOAR placement site 76 days after implantation. Antibiotic therapy and percutaneous drainage were ineffective; thus laparoscopic abscess fenestration was performed. Abscess size reduction was noted, leading to clinical improvement and a favorable course.

**Conclusion:**

This is the first known reported case of laparoscopic abscess fenestration for a SpaceOAR‐related abscess and the findings highlight the need to include abscess formation as a differential diagnosis even after more than 2 months following placement. Furthermore, this case indicates laparoscopic fenestration as a viable treatment option for a rectal spacer‐related abscess unresponsive to antibiotics or percutaneous drainage.

**Summary**

- This is the first reported case of laparoscopic fenestration for an abscess related to SpaceOAR placement.
- When antibiotic therapy and percutaneous drainage are ineffective, minimally invasive surgery can offer a safe and effective treatment option.

## 1 Introduction

Radiation therapy is an effective treatment option for localized prostate cancer, though it can lead to complications, such as rectal bleeding, diarrhea, and proctitis [1, 2]. The SpaceOAR system (Boston Scientific, Marlborough, MA, USA) is a hydrogel spacer used to reduce radiation exposure to the rectum and related adverse events (AEs). Despite evidence supporting its efficacy and feasibility, complications such as misplacement, infection, and fistula formation have been documented [3, 4]. In this report, we describe a case of abscess following SpaceOAR placement successfully treated with laparoscopic fenestration.

## 2 Case Presentation

Reported here is a case of localized prostate cancer (PSA 8.8 ng/mL, cT2aN0M0, Gleason score 3 + 4 in 1/12 cores, 3 + 3 in 5/12 cores) in a 70‐year‐old male who subsequently developed a multiloculated abscess following SpaceOAR placement.

The treatment plan included low‐dose‐rate (LDR) brachytherapy with permanent seed implantation delivering 110 Gy, along with external beam radiation therapy (EBRT) at a dose of 45 Gy in 25 fractions. Furthermore, androgen deprivation therapy with goserelin and bicalutamide was initiated 2 months before seed implantation. Under general anesthesia, the seeds were implanted first, followed by manual injection of SpaceOAR hydrogel into the rectoprostatic space.

**FIGURE 1:**
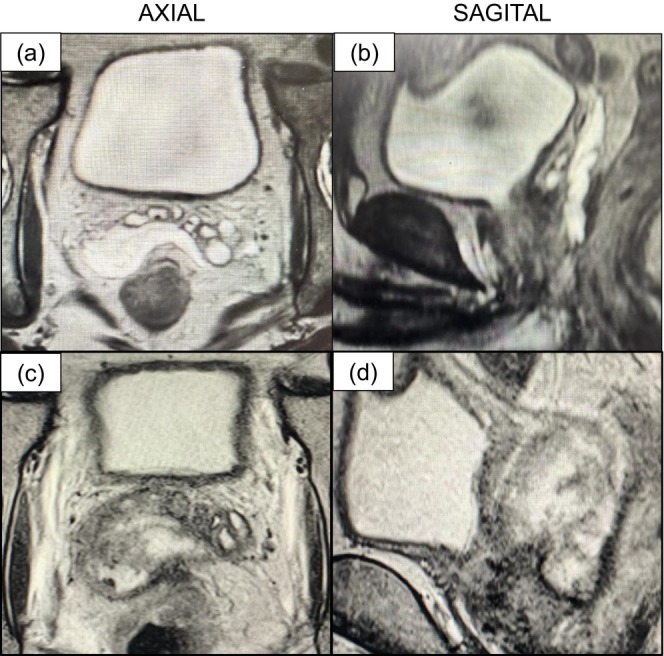
MRI images—comparison before and after infection. (a, b) MRI image after SpaceOAR placement. (c, d) MRI images at the time of infection.

Postoperative MRI performed 26 days after the procedure showed that the hydrogel was placed between the rectum and the prostate, extending cranially and located dorsal to the seminal vesicles (Figure 1). EBRT was initiated 56 days postoperatively.

On day 77, the patient developed a fever (38.2°C) and noted urinary frequency. A digital rectal examination found no tenderness, while urinalysis showed no pyuria and urine cultures were negative. However, blood testing revealed elevated WBC 14400/μL and CRP 12.12 mg/dL, indicating systemic inflammation. The planned EBRT regimen was 45 Gy in 25 fractions, though treatment was discontinued after 22 fractions due to suspected infection. Contrast‐enhanced CT and MRI revealed a multiloculated abscess at the site of SpaceOAR placement (Figure 1), as well as inflammation spreading along the hydrogel, suggesting a related infection.

**FIGURE 2:**
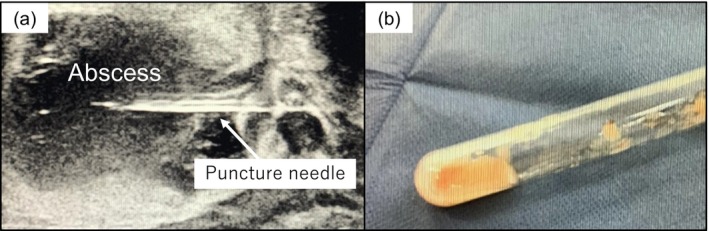
Abscess aspiration. (a) Ultrasound‐guided transperineal abscess drainage. (b) Pus retrieved from the abscess aspiration; the sample was highly viscous, and only a small volume was obtained.

Empirical treatment with levofloxacin was initiated. Nevertheless, the fever persisted, prompting escalation to meropenem, which also failed to resolve the infection. Ultrasound‐guided transperineal drainage was performed twice, though each yielded only 3 cc of thick yellowish‐white purulent fluid (Figure 2). Culturing of the aspirated fluid indicated *Finegoldia magna* , a Gram‐positive anaerobic bacterium.

**FIGURE 3:**
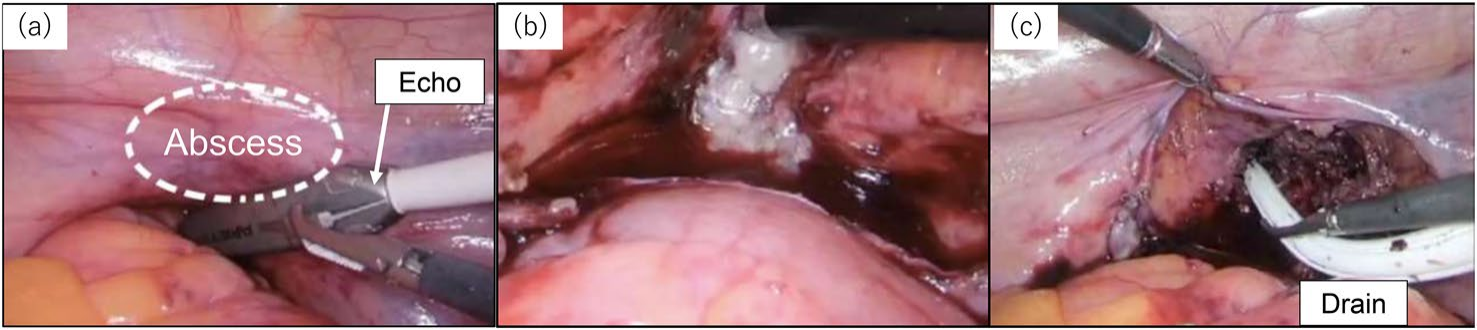
(a) Abscess drainage was performed under intraoperative ultrasound visualization. (b) Drainage of pus was observed from the pelvic floor. (c) A drain was placed, and the procedure was completed.

Given the failure of antibiotic therapy and percutaneous drainage, laparoscopic abscess fenestration was performed on day 91. The surgical procedure was performed in the lithotomy position with a 20° Trendelenburg tilt. A camera port was placed cephalad to the umbilicus, with bilateral 12‐mm ports 8 cm laterally and an additional 5‐mm assistant port lateral to the right port. The operative time was about 4 h. Intraoperatively, transrectal and intra‐abdominal ultrasound confirmed the abscess location. After peritoneal incision, whitish gelatinous pus and multiple loculated cavities were identified, irrigated, and aspirated (Figure 3). A pelvic drain was placed, and a leak test was performed to avoid bladder injury. The abscess gradually resolved following the procedure and inflammatory markers normalized. The patient was discharged on day 107 with complete resolution of symptoms. Because of the possibility of recurrent infection, EBRT was not resumed. Although EBRT could not be completed, follow‐up after discharge has shown no biochemical recurrence.

## 3 Discussion

Rectal toxicity is a specific complication associated with radiation therapy for localized prostate cancer and can cause rectal bleeding, proctitis, mucus discharge, tenesmus, and fecal incontinence. Grade 2 or higher rectal toxicity is estimated to occur in 5%–15% of treated patients, significantly impacting the quality of life [5, 6].

An advanced technique for patients undergoing prostate cancer radiation therapy involves the insertion of a hydrogel rectal spacer between the prostate and rectum, an approach that reduces the risk of radiation exposure to the latter, effectively mitigating or avoiding rectal toxicity [5]. Nevertheless, various AEs associated with SpaceOAR, a commonly used hydrogel spacer, have been reported. A previous review of the Manufacturer and User Facility Device Experience database found approximately 200 000 cases of SpaceOAR placement from 2015 to 2023 [7]. Among those, 990 cases with AEs were documented, including 91 with abscesses, 91 with fistulas, 79 with infections, and 71 with ulcers related to the use of the spacer. Additionally, five cases with a fatal outcome were reported. Of the 332 cases with AEs, the timing of radiation therapy in relation to the infection was noted in 156, with 35 (22.4%) occurring before radiation therapy, and 121 (77.6%) during or after radiation therapy, showing a significantly higher incidence rate related to radiation therapy.

At our institution, the clinical target volume was the prostate plus seminal vesicles, with a 6‐mm margin for the planning target volume. EBRT was delivered using four‐field 3D‐CRT. CT/MRI at 4 weeks after seed implantation and the CT before EBRT showed no abscess. Although part of the abscess extended outside the field, the portion dorsal to the seminal vesicles was within the high‐dose region. These findings suggest that abscess formation had not yet occurred at that time. In the present patient, 76 days had passed since SpaceOAR placement, and infection occurred following initiation of EBRT, which is known to cause lymphedema due to necrosis, granulation, and/or fibrosis of micro‐lymphatic vessels and surrounding soft tissues. It was therefore considered that the presence of the hydrogel might have contributed to abscess formation, and its involvement was included in the differential diagnosis, even though more than 2 months had passed since placement.

A previous study reported that surgical intervention was required in 60 cases with perineal abscesses or vesicorectal fistulas, which involved such procedures as abscess drainage or creation of a colostomy, while it was also noted that surgical drainage has been shown to shorten the duration of antibiotic use, reduce hospitalization time, and improve voiding function [8]. Although our case could not be managed with a single puncture, transperineal pig‐tail catheter placement may also represent a therapeutic option [9].

To our knowledge, this is the first report of a SpaceOAR‐related abscess refractory to antibiotics and percutaneous drainage, successfully managed with laparoscopic fenestration. This approach may be considered a viable treatment option for abscesses unresponsive to conventional therapy or drainage techniques.

## 4 Conclusion

Laparoscopic fenestration was used to successfully treat a refractory SpaceOAR‐related abscess unresponsive to antibiotics or percutaneous drainage. This case highlights the importance of considering severe complications associated with the use of rectal spacers and suggests laparoscopic fenestration as a viable treatment option. Further research is necessary for the establishment of optimal management strategies for such complications.

## Consent

The authors have nothing to report.

## Conflicts of Interest

The authors declare no conflicts of interest.

## Data Availability

Data sharing not applicable to this article as no datasets were generated or analyzed during the current study.

## References

[1] R. Jadon , E. Higgins , L. Hanna , M. Evans , B. Coles , and J. Staffurth , “A Systematic Review of Dose‐Volume Predictors and Constraints for Late Bowel Toxicity Following Pelvic Radiotherapy,” Radiation Oncology 14, no. 1 (2019): 57, 10.1186/s13014-019-1262-8. 30943992 PMC6448293

[2] D. Y. Joh , L. N. Chen , G. Porter , et al., “Proctitis Following Stereotactic Body Radiation Therapy for Prostate Cancer,” Radiation Oncology 9 (2014): 277, 10.1186/s13014-014-0277-4. 25497602 PMC4272823

[3] U. Azhar , J. Lin , R. Sayed , Z. Masoud , A. Zamarud , and R. Kaler , “Perineal Abscess Following SpaceOAR Insertion,” Cureus 15, no. 12 (2023): e51050, 10.7759/cureus.51050. 38146336 PMC10749505

[4] J. M. Kuperus , D. G. Kim , T. Shah , G. Ghareeb , and B. R. Lane , “Rectourethral Fistula Following SpaceOAR Gel Placement for Prostate Cancer Radiotherapy: A Rare Complication,” Urology Case Reports 35 (2020): 101516, 10.1016/j.eucr.2020.101516. 33318943 PMC7726656

[5] M. Babar , A. Katz , and M. Ciatto , “Dosimetric and Clinical Outcomes of SpaceOAR in Men Undergoing External Beam Radiation Therapy for Localized Prostate Cancer: A Systematic Review,” Journal of Medical Imaging and Radiation Oncology 65, no. 3 (2021): 384–397, 10.1111/1754-9485.13179. 33855816

[6] E. J. Lehrer , A. U. Kishan , J. B. Yu , et al., “Ultrahypofractionated Versus Hypofractionated and Conventionally Fractionated Radiation Therapy for Localized Prostate Cancer: A Systematic Review and Meta‐Analysis of Phase III Randomized Trials,” Radiotherapy and Oncology 148 (2020): 235–242, 10.1016/j.radonc.2020.04.037. 32505965

[7] J. C. Millot , C. Arenas‐Gallo , E. Silver , et al., “Major Complications and Adverse Events Related to Use of SpaceOAR Hydrogel for Prostate Cancer Radiotherapy,” Urology 188 (2024): 94–100, 10.1016/j.urology.2023.12.034. 38458325

[8] D. S. Lee , H. S. Choe , H. Y. Kim , et al., “Acute Bacterial Prostatitis and Abscess Formation,” BMC Urology 16 (2016): 38, 10.1186/s12894-016-0153-7. 27388006 PMC4936164

[9] D. C. Sperling , L. Needleman , D. J. Eschelman , D. M. Hovsepian , and A. S. Lev‐Toaff , “Deep Pelvic Abscesses: Transperineal US‐Guided Drainage,” Radiology 208, no. 1 (1998): 111–115, 10.1148/radiology.208.1.9646800. 9646800

